# Increased CO_2_ Relevant to Future Ocean Acidification Alleviates the Sensitivity of a Red Macroalgae to Solar Ultraviolet Irradiance by Modulating the Synergy Between Photosystems II and I

**DOI:** 10.3389/fpls.2021.726538

**Published:** 2021-09-16

**Authors:** Di Zhang, Juntian Xu, Sven Beer, John Beardall, Cong Zhou, Kunshan Gao

**Affiliations:** ^1^State Key Laboratory of Marine Environmental Science & College of Ocean and Earth Sciences, Xiamen University, Xiamen, China; ^2^Jiangsu Key Laboratory of Marine Bioresources and Environment, Jiangsu Ocean University, Lianyungang, China; ^3^Department of Plant Sciences and Food Security, Faculty of Life Sciences, Tel Aviv University, Tel Aviv, Israel; ^4^School of Biological Sciences, Monash University, Clayton, VIC, Australia

**Keywords:** chlorophyll fluorescence, CO_2_ enrichment, ocean acidification, photosystems II and I, photoinhibition, *Pyropia yezoensis*, ultraviolet-radiation

## Abstract

While intertidal macroalgae are exposed to drastic changes in solar photosynthetically active radiation (PAR) and ultraviolet radiation (UVR) during a diel cycle, and to ocean acidification (OA) associated with increasing CO_2_ levels, little is known about their photosynthetic performance under the combined influences of these drivers. In this work, we examined the photoprotective strategies controlling electron flow through photosystems II (PSII) and photosystem I (PSI) in response to solar radiation with or without UVR and an elevated CO_2_ concentration in the intertidal, commercially important, red macroalgae *Pyropia* (previously *Porphyra*) *yezoensis*. By using chlorophyll fluorescence techniques, we found that high levels of PAR alone induced photoinhibition of the inter-photosystem electron transport carriers, as evidenced by the increase of chlorophyll fluorescence in both the J- and I-steps of Kautsky curves. In the presence of UVR, photoinduced inhibition was mainly identified in the O_2_-evolving complex (OEC) and PSII, as evidenced by a significant increase in the variable fluorescence at the K-step (*F*_k_) of Kautsky curves relative to the amplitude of *F*_J_−*F*_o_ (W_k_) and a decrease of the maximum quantum yield of PSII (*F*_v_/*F*_m_). Such inhibition appeared to ameliorate the function of downstream electron acceptors, protecting PSI from over-reduction. In turn, the stable PSI activity increased the efficiency of cyclic electron transport (CET) around PSI, dissipating excess energy and supplying ATP for CO_2_ assimilation. When the algal thalli were grown under increased CO_2_ and OA conditions, the CET activity became further enhanced, which maintained the OEC stability and thus markedly alleviating the UVR-induced photoinhibition. In conclusion, the well-established coordination between PSII and PSI endows *P. yezoensis* with a highly efficient photochemical performance in response to UVR, especially under the scenario of future increased CO_2_ levels and OA.

## Introduction

Living in the intertidal zone, macroalgae are often exposed to periodic harsh light fluctuations and air exposure associated with changes in tide levels. High levels of solar irradiance can significantly decrease photosynthesis and growth rates in macroalgae (Aline et al., 2006; Martin and Gattuso, 2009; Ji and Gao, 2020), while limited light would entail an insufficient energy supply and thus decrease photosynthesis and growth. Under limited light conditions, longer wavelengths within the range of ultraviolet radiation (UVR, 280–400nm), generally considered to be detrimental to aquatic ecosystems, can be used as light energy for photosynthesis (Gao et al., 2007). Moderate levels of UVA (315–400nm) are beneficial for carbon fixation in several macroalgae (Gao and Xu, 2008; Xu and Gao, 2010), and can also act as a signal to stimulate the activity of carbonic anhydrase and nitrate reductase (Viñegla et al., 2006), or prompt morphological development during germination of conchospores (Jiang et al., 2007). Furthermore, the effects of UVR also depend strongly on interactions with other environmental factors. For example, increased ocean temperatures result in stratification and shoaling of the upper mixed layer and thus expose organisms to increased levels of solar photosynthetically active radiation (PAR) and UVR (Häder and Barnes, 2019 and reference therein), and the global warming-induced melting of ice and snow would also aggravate the transmission of UVR and increase UVR exposure in polar regions (Williamson et al., 2019; Neale et al., 2021 and references therein). These interactive effects control the levels of exposure of macroalgae to UVR, and may modulate their photosynthetic performance, production of photoprotective compounds and/or repair mechanisms in response to UVR (see the review by Ji and Gao, 2020 and references therein).

As a consequence of anthropogenic CO_2_ emissions, the atmospheric CO_2_ concentration has been predicted to increase to above 1,000 μatm by the end of this century (e.g., IPCC, 2014). In addition to possible direct effects of higher aqueous CO_2_, this will also result in an increase in proton concentration in the seawater (a drop in pH from 8.1 to 7.8), known as ocean acidification (OA). A number of previous studies have shown that OA hindered calcification processes (Gao et al., 1993; Semesi et al., 2009; Gao and Zheng, 2010; Büdenbender et al., 2011) and thus exposed calcified algae to more UVR exposure. In contrast, the elevated availability of dissolved inorganic carbon (DIC) in seawater has been reported to stimulate both photosynthesis and growth in a number of non-calcified macroalgae such as in *Pyropia* sp. (Gao et al., 1991; Zhang et al., 2020), *Palmaria* sp. (Beer and Koch, 1996), *Gloiopeltis* sp., *Gigartina* sp. (Zou and Gao, 2005), *Gracilaria* sp. (Andría et al., 1999, 2001), *Hypnea* sp. (Suárez-Álvarez et al., 2012), and *Ellisolandia* sp. (Korbee et al., 2014). In addition, the increased DIC would also down-regulate the CO_2_-concentrating mechanisms (CCMs), which utilize HCO_3_^−^ to compensate for the limitation of CO_2_ in seawater and maintain high intracellular CO_2_ levels for photosynthesis and growth of the macroalgae (e.g., a green algae *Ulva prolifera* in Xu and Gao, 2012, and a red algae *Pyropia yezoensis* in Li et al., 2016). Since down-regulation of CCMs is known to save operational energy cost (Raven et al., 2014 and references therein), the energy savings can either stimulate algal growth under low light and increase the risk of photoinhibition under high light (especially with the presence of UVR; see the review by Gao et al., 2019 and references therein).

*Pyropia* (previously known as *Porphyra*; Rhodophyta), an economically important marine crop worth ~US$1.3 billion per year (Blouin et al., 2011), has been widely cultivated in both China and other Asian countries. Previously, we showed that UVR inhibited both carbon assimilation and growth of *P. yezoensis*, while elevated CO_2_ exhibited a positive effect and participated in the alleviation of the UVR-induced inhibition (Zhang et al., 2020). In that work, increases of non-photochemical quenching (NPQ) and UV-absorbing compounds (UVACs) were suggested to dissipate and/or absorb the excess energy originating from UVR, while little attention was paid to the transfer of such absorbed energy. In red algae, phycobilisomes (PBS) form the light-harvesting antennae on the outer surface of thylakoid membranes, in the proximity of photosystem II (PSII), the specific mechanisms for this are unclear but may involve state transitions or mobility of PBS, redistributing the energy between the two photosystems and thus altering photosynthetic electron transport and supply of energy for CO_2_ fixation and reduction (Su et al., 2010 and references therein). Moreover, regulation of photosynthetic electron transport, e.g., *via* alternative electron transport chains, including cyclic electron transport (CET) around photosystem I PSI, photorespiration and the water-water cycle along with reactive oxygen species (ROS)-scavenging systems, has also been supposed to protect photosynthetic systems from photoinhibition/photodamage (Eberhard et al., 2008 and references therein, Miyake, 2010). In *P. yezoensis*, CET has been verified to play a vital role in photoprotection when thalli suffered from dehydration (Gao and Wang, 2012), severe salt stress (Lu et al., 2016; Yu et al., 2018), and irradiance stress (Niu et al., 2016). The active CET not only participates in NPQ, but also alleviates the over-reduction of plastoquinone and, thus, balance the redox state of the photosynthetic electron transport chain (Miyake, 2010).

In the present study, effects of OA and UVR on the photosynthetic performance of *P. yezoensis* were investigated by growing these algae under incident solar radiation with or without UVR at ambient and elevated CO_2_ concentrations projected for future OA by the end of 2100. While high CO_2_ and the concomitant OA may have separate effects on algal physiology in nature (Hurd et al., 2020), technically, it is hard to distinguish the specific effects of pH or CO_2_. Moreover, pH and CO_2_ covary oppositely even in algal blooms or with progressive OA, thus we did not attempt to disentangle the interactions between these two variables. Our aims are 1) characterized the electron transport flux from PSII to PSIl, 2) examined an alternative electron sink, i.e., CET; and 3) evaluated the coordination between PSII and PSI, under the influences of UVR and OA.

## Materials and Methods

### Experimental Treatments and Measurements of UV Irradiance and pH

Thalli of *P. yezoensis* (Ueda) M.S.Hwang & H.G.Choi were collected from rafts offshore of Gaogong Island (34°43′31′ N, 119°31′57′ E), Lianyungang, Jiangsu Province, China, on December 12, 2017, and transported to the laboratory in a cooled Styrofoam box within 2h. Following rinsing, thalli of ~0.05*g* fresh weight were grown outdoors for 9days in 1L open-ended quartz tubes filled with natural seawater, which were partly immersed in a flow-through water bath to maintain the seawater temperature at 8±1°C. The seawater in each tube was continuously aerated (300ml per min) with air containing 400±20 or 1,000±50 μatm CO_2_, and was renewed every day. The low-CO_2_ air was directly obtained with an air pump while the high-CO_2_ level was obtained from a CO_2_ enricher (HP 1000G-D, Ruihua Instruments, Wuhan, China), which controls the CO_2_ concentration with less than 5% variation. Different radiation treatments were achieved by covering the quartz tubes with Ultraphan film 395 (UV Opak, Digefra, Munich, Germany), Folex 320 film (Montagefolie, Folex, Dreieich, Germany), or Ultraphan film 295 (Digefra), respectively, so that the thalli were exposed to irradiances above 395nm (PAR alone), above 320nm (PA, PAR+UVA) and above 295nm (PAB, PAR+UV-A+B), respectively. Considering the low density of algal blades in the tubes, the self-shading in our present study can be considered minimal. Measurements of photochemical activities (see below) were carried out around 14:00 on the 10th day of treatments. A total of 18 tubes containing different individual thalli were used for measurements, and three independent thalli were used as replicates for each parameter. According to published papers (Mercado et al., 1999; Zou, 2005; Chen et al., 2016, 2017), and also based on our previous experience (Zou et al., 2003; Xu and Gao, 2008, 2010), 10days culture is enough for full acclimation of the photosynthetic and other biochemical traits in *Pyropia* spp. and other tested marine macroalgae.

The pH_NBS_ was measured at the end of each day by a pH meter (pH 700, Eutech Instruments, Singapore) equipped with an Orion^®^ 8102BN Ross combination electrode (Thermo Electron Co., United States), which was calibrated with NBS standard buffers every day during the experiment (Thermo Fisher Scientific Inc., United States). Total alkalinity (TA) was measured with a TA analyzer (AS-ALK1, Apollo SciTech, United States) by Gran acidimetric titrations. The values of other carbonate chemistry parameters (total inorganic carbon concentration, TIC, bicarbonate and carbonate ions) were calculated by the Excel program CO2SYS (Pierrot et al., 2006) according to the measured values of TA and pH_NBS_.

The incident solar irradiances were continuously monitored and recorded every minute by a broadband solar radiometer (EKO Instruments Co., LTD, Japan), which has three separate channels, for (PAR, 400–700nm), UVA (315–400nm), and UVB (280–315nm), respectively.

Before the final measurements, the *in situ* diurnal variations (daytime) of pH and CO_2_ were measured. These results showed that the total alkalinity (TA) was around ~2,400μm throughout the day, pH ranged from ~8.2 to 8.4 and the dissolved CO_2_ ranged from about 10 to 13μm. The maximal and daily average PAR values during the experimental period were 812.6±57.4 and 186.1±35.1μmol photons m^−2^ s^−1^, respectively, while the corresponding values for UVA were 8.1±0.7 and 1.9±0.3Wm^−2^, and that for UVB 0.3±0.03 and 0.1±0.01Wm^−2^. PAR, UVA and UVB levels were 635μmol photons m^−2^ s^−1^ and 6.5 and 0.2Wm^−2^, respectively, when the following parameters were measured at 14:00 on the 10th day. During the experiment, the enhanced CO_2_ level (from 400 to 1000μatm in the air phase) resulted a pH drop from 8.24±0.03 to 7.92±0.03 (*n*=27). While TA remained unaltered, the TIC increased from 2,131±20 to 2,310±20μm and that of CO_2_ from 12±1 to 28±3μm (*n*=27) under the high-CO_2_ treatment.

### Chlorophyll Fluorescence Measurements and Analyses

A dual-wavelength pulse-amplitude-modulated (PAM) fluorescence monitoring system (Dual-PAM-100, Walz, Effeltrich, Germany) was employed to simultaneously measure the performance of PSII and PSI. To avoid the effect of phycobiliproteins on chlorophyll fluorescence, blue light (440nm) was used as excitation light in the following measurements. Rapid fluorescence induction kinetics (Kautsky curves) showed a typical polyphasic rise pattern between O (the minimum fluorescence) and P (the maximum fluorescence) during the first second of illumination (Neubauer and Schreiber, 1987). The typical Kaustsky curve plotted against a logarithmic time scale represented different processes of photosynthetic electron transport (Supplementary Figure S1). According to Strasser and Strasser (1995) and Guisse et al. (1995), the fluorescence characterized of several different phases, where the time-specific steps were labeled as O, K (at ~300μs), J (at ~2ms), I (at ~30ms) and P. Fluorescence intensities at different phases were noted as *F*_o_, *F*_k_, *F*_J_, *F*_I_ and *F*_m_. The standardized fluorescence intensity from the O- to P-phase was calculated as *V*_t_=(*F*_t_−*F*_o_)/(*F*_m_−*F*_o_). To assess the donor side activity of PSII, the normalized variable fluorescence at the K-step relative to the amplitude of *F*_J_−*F*_o_ (*W*_k_) was calculated as *W*_k_=(*F*_k_−*F*_o_)/(*F*_J_−*F*_o_). To evaluate the activity of PSII, the maximum quantum yield of PSII (*F*_v_/*F*_m_) was calculated as *F*_v_/*F*_m_=(*F*_m_−*F*_o_)/*F*_m_. The acceptor side activity of PSII, i.e., the probability that a trapped exciton moves an electron into the electron transport chain beyond Q_A_^−^ ($\psi_{\mathrm{ET}2O}$) and the quantum yield of electron transport (φ_Eo_) was calculated as $\psi_{\mathrm{ET}2O}$=1−*V*_J_ and φ_Eo_=(1−*F*_o_/*F*_m_)×(1−*V*_J_), respectively. The redox state of inter-photosystem electron carriers and the acceptor side activity of PSI, i.e., the probability that an electron moves from reduced Q_A_ beyond PSI ($\psi_{\mathrm{RE}1O}$), and the quantum yield for reduction of the end electron acceptors on the PSI acceptor side (φ_Ro_), were calculated as $\psi_{\mathrm{RE}1O}$=1−*V*_I_ and φ_Ro_=(1-*F*_o_/*F*_m_)×(1−*V*_I_), respectively. All these parameters were derived from JIP-tests (Strasser and Strasser, 1995; Strasser et al., 2004). According to the theory of energy fluxes in biomembranes (Strasser, 1981), the density of the PSII reaction center per excited cross section (RC/CSo), the absorbed flux (ABS), the trapping flux (TRo), the electron transport flux (ETo), and the dissipated energy flux (DIo) by active reaction centers were calculated as.

$$\mathrm{RC}/\mathrm{CSo}=F_{v}/F_{m}\times V_{J}/V_{K}/4\times F_{O},$$

$$\mathrm{ABS}/\mathrm{RC}=4\times \left(F_{K}-F_{O}\right)\times F_{m}/\left(F_{J}-F_{O}\right)\times F_{v},$$

$$\mathrm{TRo}/\mathrm{RC}=4\times \left(F_{K}-F_{O}\right)/\left(F_{I}-F_{O}\right),$$

$$\mathrm{ETo}/\mathrm{RC}=4\times \left(F_{K}-F_{O}\right)\times \left(F_{m}-F_{J}\right)/\left(F_{J}-F_{O}\right)\times F_{v}$$

and

DIo/RC=ABS/RC−TRo/RC.

### P700 Measurements and Analyses

As suggested by Klughammer and Schreiber (1994), the P700^+^ signal measured with the dual-wavelength (830/875nm) unit of the instrument was taken as a measure of the redox state of P700. After 10s exposure to far-red light, a saturation flash was applied to determine the maximum P700^+^ signal (*P*_m_). The steady-state P700^+^ signal (*P*) was monitored under actinic light generated by the instrument at similar PAR levels as the natural sunlight (~800μmol photons m^−2^ s^−1^). The 0.8s saturating flash of ~10, 000μmol photons m^−2^ s^−1^ was applied to induce the maximum P700^+^ value (*P*_m_′). The effective quantum yield of PSI (YI) was calculated as (*P*_m_′−*P*)/*P*_m_.

In *Pyropia* spp., several published papers have demonstrated that the CET around PSI could account for up to 97.7% of total electron flow when algal blades suffered from severe desiccation (Gao and Wang, 2012). This was thus supposed to be one of the most important alternative electron transport pathway during exposure to stresses (Gao et al., 2013; Yu et al., 2018). Accordingly, our present study paid more attention on the physiological role of CET during the exposure to UVR and high-CO_2_ induced OA. CET around PSI was evaluated by the measurement of the re-reduction kinetics of P700^+^. After ~10s exposure to far-red light, the applied saturation flash drives P700^+^ to combine with electrons, and the initial linear slope of the re-reduction of P700^+^ indicated the activity of CET.

All measured and calculated parameters are summarised in Table 1.

**Table 1:** Measured and calculated parameters used in this paper.

Parameters	Physiological interpretation
Kautsky curves	
*F*_o_, *F*_k,_*F*_J_, *F*_I_, *F*_P_	fluorescence intensity at O, K, J, I, P phases
*V*_t_	relative variable fluorescence at time t
*W*_k_=(*F*_k_−*F*_o_)/(*F*_J_−*F*_o_)	normalized variable fluorescence at the K-step relative to the amplitude of *F*_J_−*F*_o_
*F*_v_/*F*_m_=(*F*_m_−*F*_o_)/*F*_m_	maximum photochemical efficiency of PSII
RC/CS_o_=*F*_v_/*F*_m_×*V*_J_/*V*_K_/4×*F*_o_	density of PSII RC per excited cross sections
$\psi_{\mathrm{ET}2O}$=1−*V*_J_	probability that a trapped excition moves an electron into the electron transport chain beyond Q_A_^−^
φ_Eo_=(1−*F*_o_/*F*_m_)×(1−*V*_J_)	quantum yield of electron transport
$\psi_{\mathrm{RE}1O}$=1−*V*_I_	probability that an electron moves from reduced Q_A_ beyond PSI
φ_Ro_=(1−*F*_o_/*F*_m_)×(1−*V*_I_)	quantum yield for reduction of the end electron acceptors on the PSI acceptor side
ABS/RC=4×(*F*_k_−*F*_o_)×*F*_m_/(*F*_J_−*F*_o_)×*F*_v_	absorbed flux by active RCs
TR_o_/RC=4×(*F*_k_−*F*_o_)/(*F*_I_−*F*_o_)	trapping flux by active RCs
ET_o_/RC=4×(*F*_k_−*F*_o_)×(*F*_m_−*F*_J_)/(*F*_J_−*F*_o_)×*F*_v_	electron transport flux by active RCs
DI_o_/RC=ABS/RC−TR_o_/RC	dissipated energy flux by active RCs
P700 measurements	
*P*, *P*_m_, *P*_m_′	real-time, maximum, and maximum steady state, absorption signal of P700^+^
YI=(*P*_m_′−*P*)/*P*_m_	effective photochemical quantum yield of PSI
P700^+^ re-reduction	the activity of cyclic electron transport (CET) around PSI

PSII, photosystem II; PSI, photosystem I; RC, reaction center; CS, cross section.

### Statistical Analyses

In the present study, UVR-induced inhibition for a particular parameter was calculated as (P_PAR_−P_PAR_+_UVR_)/P_PAR_×100%, where P_PAR_ and P_PAR_+_UVR_ represent the values of the physiological parameter for the thalli grown under PAR alone or PAR+UVR, respectively. UVB-induced inhibition was derived from the difference in the values between the PAB (PAR+UVA+B) and PA (PAR+UVA) treatments.

Statistical analyses were performed using SPSS 19.0 (SPSS Inc., Chicago, IL, United States). The homogeneity of variance was examined using Levene’s test before all statistical analyses. One-way ANOVA and *t*-test were used to establish differences among treatments. A two-way ANOVA was used to identify the effects of CO_2_ concentration, light, UV, and their interactions. Differences were considered to be statistically significant at *p*<0.05.

## Results

**Figure 1:**
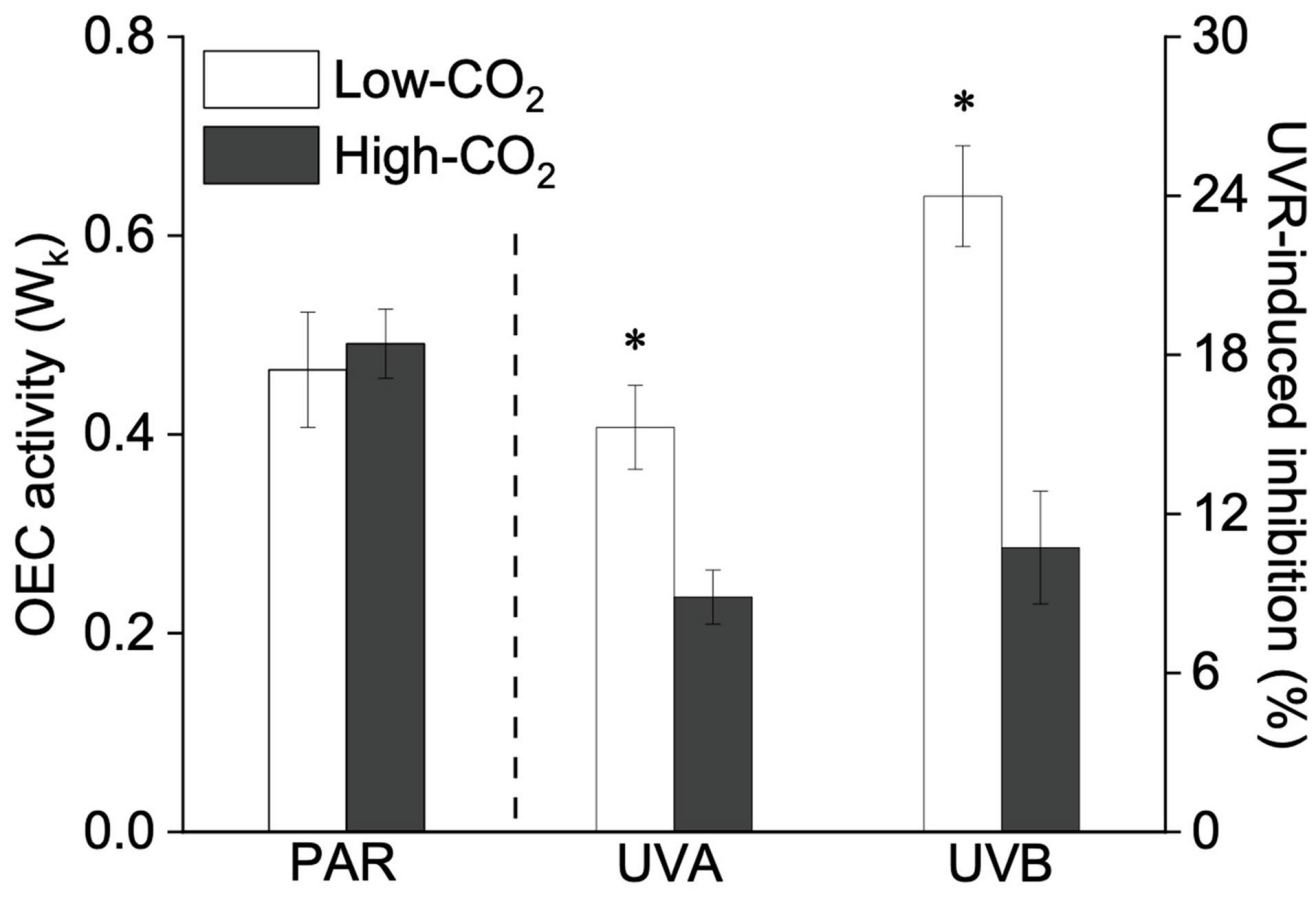
Values (a.u., left pair of bars) and UVR-induced inhibition (%, right two pairs of bars) on the O_2_-evolving complex (OEC) activity (the normalized variable fluorescence at the K-step relative to the amplitude of *F*_J_−*F*_o_, *W*_k_) of *Pyropia yezoensis* growing for 9days at low (~400μatm, open bars) and high (~1000μatm, closed bars) CO_2_ conditions. Data are means±*SD* (*n*=3). The symbol “^*^” indicates a significant (*p*<0.05, *t*-tests) difference between the treatments in each pair.

**Figure 2:**
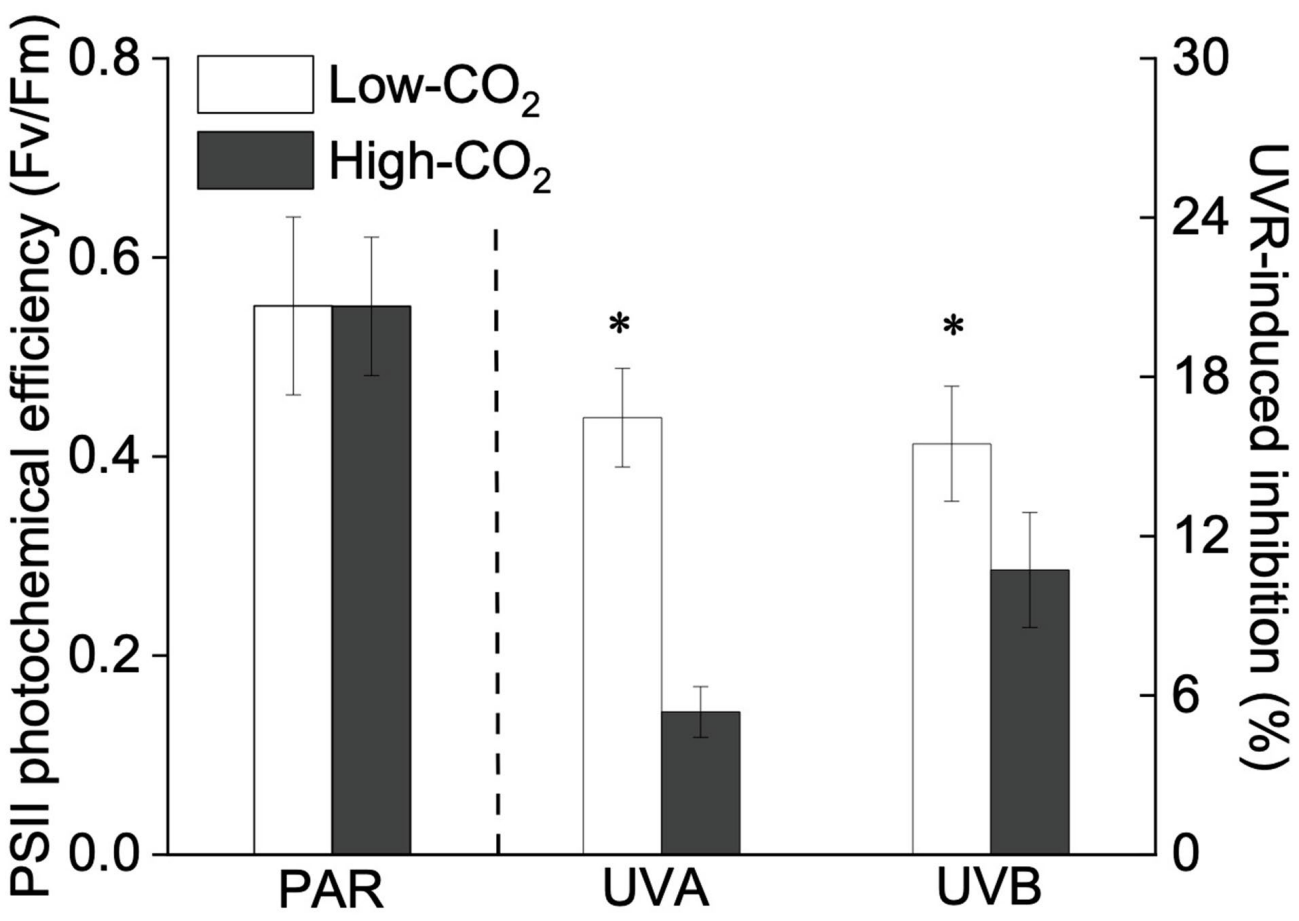
Values (a.u., left pair of bars) and UVR-induced inhibition (%, right two pairs of bars) of PSII photochemical efficiency (*F*_v_/*F*_m_) of *P. yezoensis* growing for 9days at low (~400μatm, open bars) and high (~1000μatm, closed bars) CO_2_ conditions. Data are means±*SD* (*n*=3). The symbols “^*^” indicates a significant (*p*<0.05, *t*-tests) difference between the treatments in each pair.

**Figure 3:**
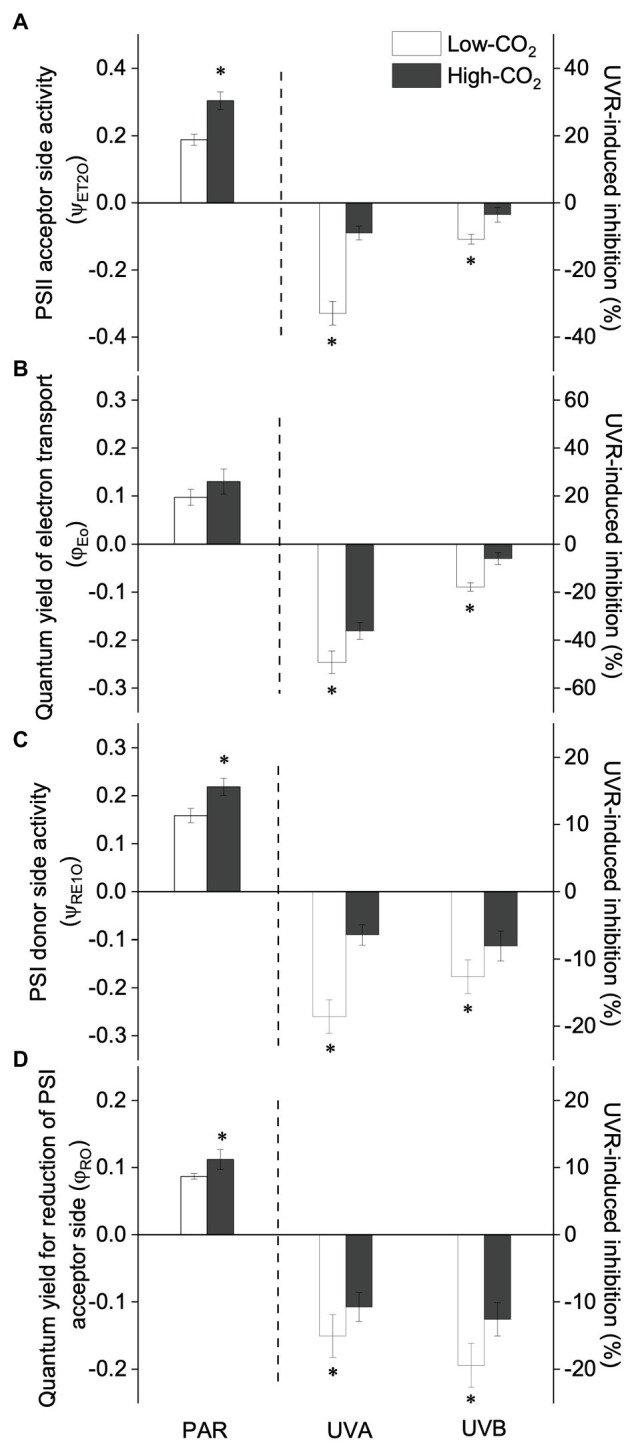
Values (a.u., left pair of bars) and UVR-induced inhibition (%, right two pairs of bars) of PSII acceptor side activity [the probability that trapped excitons move electrons into the electron transport chain beyond Q_A_^−^, $\psi_{\mathrm{ET}2O}$, panel **(A)**], quantum yield of electron transport [φ_Eo_, panel **(B)**], PSI donor side activity [the probability that an electron moves from reduced Q_A_ beyond PSI, $\psi_{\mathrm{RE}1O}$, panel **(C)**] and the quantum yield for reduction of PSI acceptor side [φ_Ro_, panel **(D)**] of *P. yezoensis* growing for 9days at low (~400μatm, open bars) and high (~1000μatm, closed bars) CO_2_ conditions. Data are means±*SD* (*n*=3). The symbols “^*^” indicates a significant (*p*<0.05, *t*-tests) difference between the treatments in each pair.

**Figure 4:**
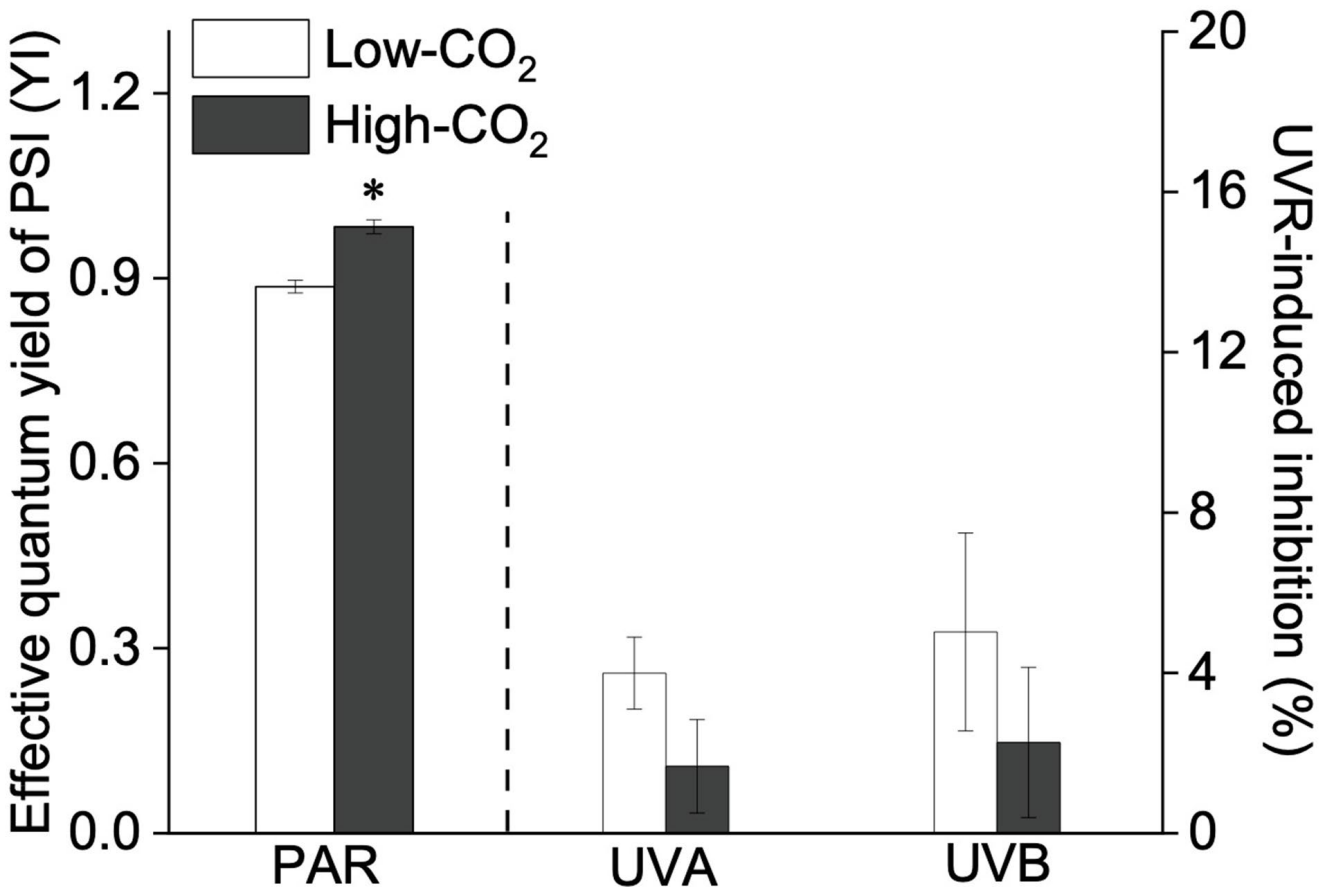
Values (a.u., left pair of bars) and UVR-induced inhibition (%, right two pairs of bars) of the effective quantum yield of PSII (YI) of *P. yezoensis* growing for 9days at low (~400μatm, open bars) and high (~1000μatm, closed bars) CO_2_ conditions. Data are means±*SD* (*n*=3). The symbols “^*^” indicates a significant (*p*<0.05, *t*-tests) difference between the treatments in each pair.

**Figure 5:**
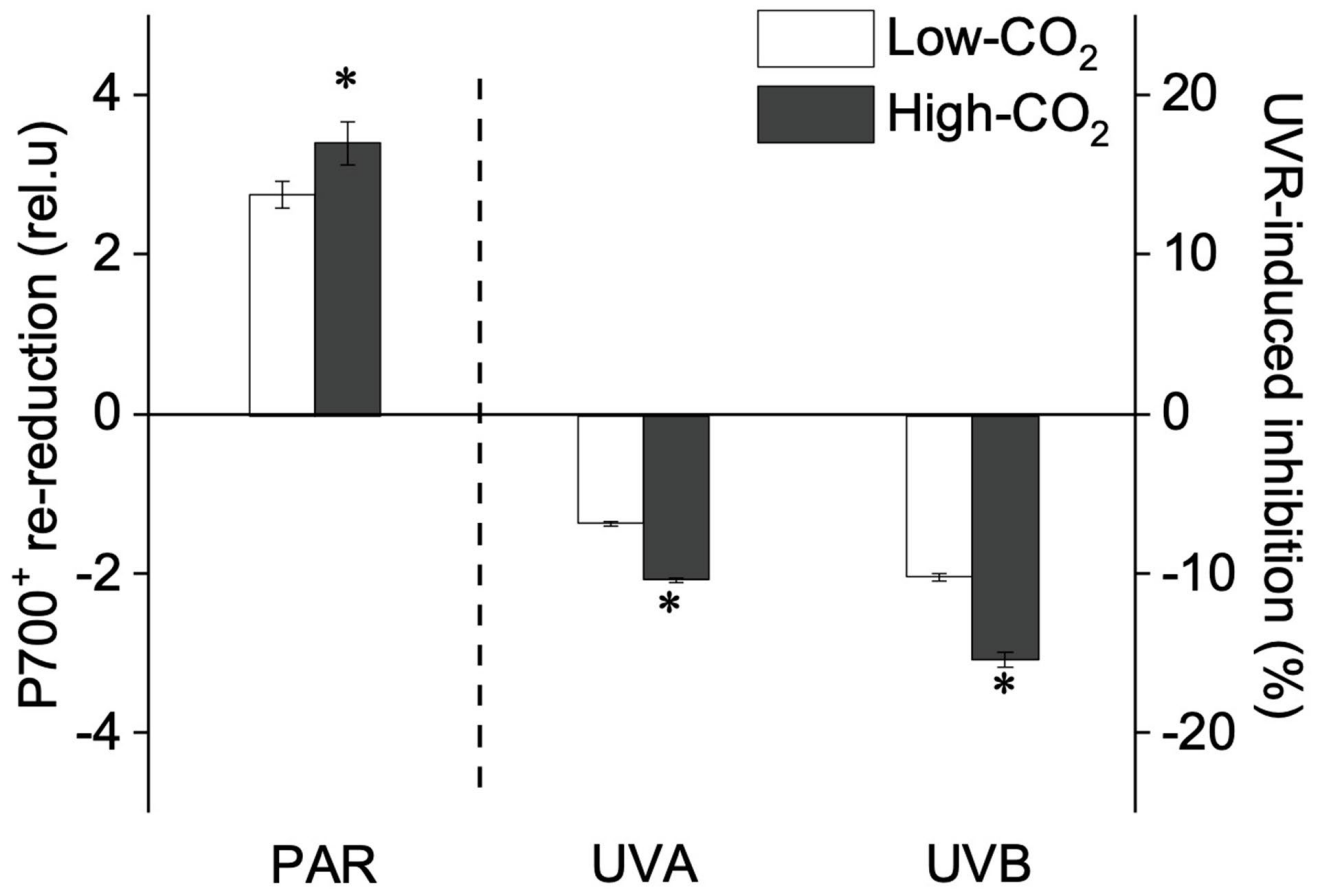
Values (a.u., left pair of bars) and UVR-induced inhibition (%, right two pairs of bars) of CET activity (the P700^+^ re-reduction rate) of *P. yezoensis* growing for 9days at low (~400μatm, open bars) and high (~1000μatm, closed bars) CO_2_ conditions. Data are means±*SD* (*n*=3). The symbols “^*^” indicates a significant (*p*<0.05, *t*-tests) difference between the treatments in each pair.

**Figure 6:**
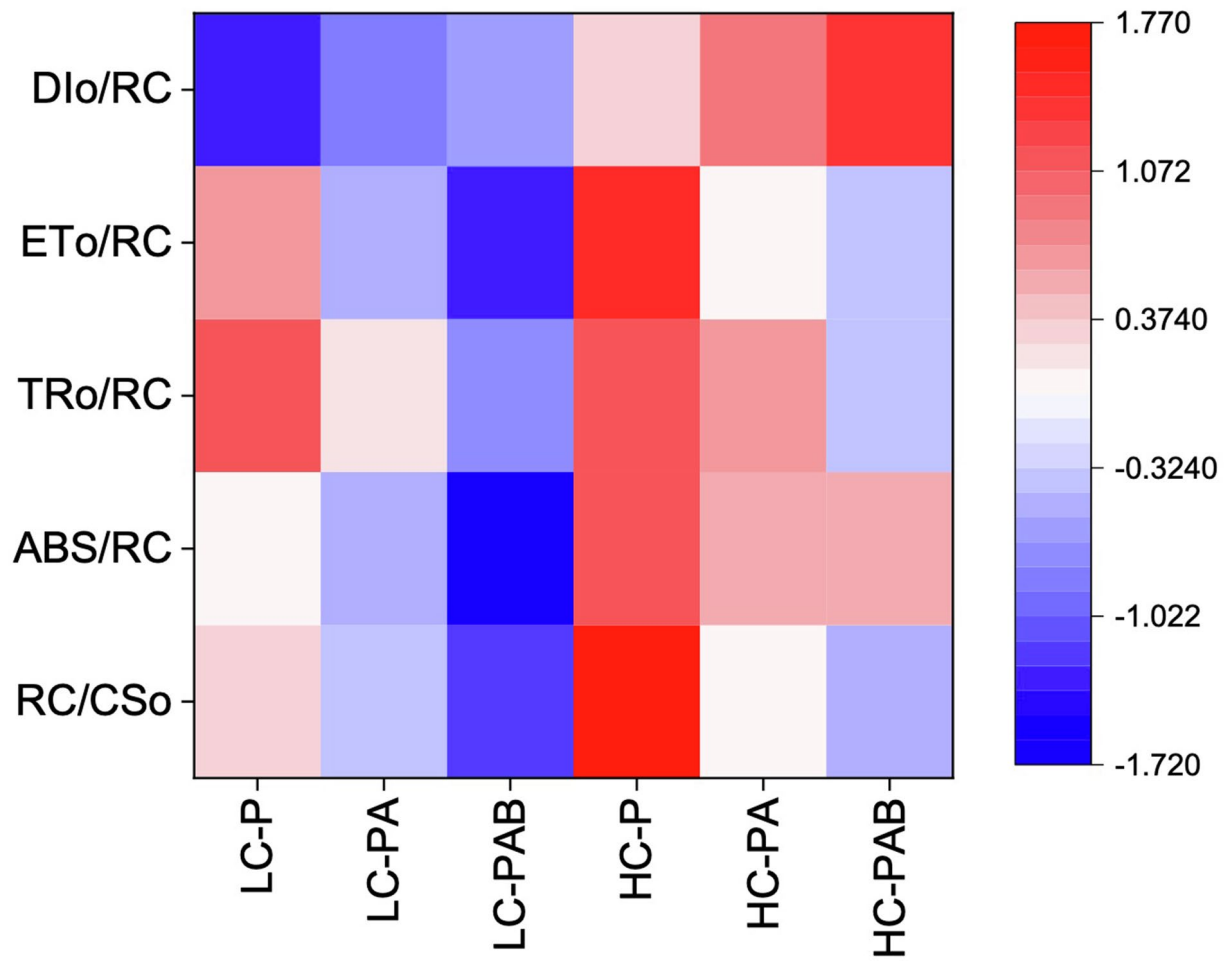
Variations in the density of the PSII reaction centers per excited cross section (RC/CSo) and the specific energy fluxes [the absorbed flux (ABS/RC), the trapping flux (TRo/RC), the electron transport flux (ETo/RC), and the dissipated energy flux (DIo/RC)] of *P. yezoensis* growing for 9days. LC and HC stand for low (~400μatm) and high (~1000μatm) CO_2_ conditions, respectively. P, PA, and PAB stand for PAR only, PAR+UVA, and PAR+UVA+UVB, respectively.

Under the ambient CO_2_ conditions (low-CO_2_), the presence of UVR significantly inhibited the O_2_-evolving complex (OEC) of PSII activities as evidenced by an increase of the variable fluorescence at the K-step of the Kautsky curve relative to the amplitude of *F*_J_−*F*_O_ (*W*_k_; *t*-test, *p*<0.05; Figure 1) and a decrease of the maximum quantum yield of PSII (*F*_v_/*F*_m_; *t*-test, *p*<0.05; Figure 2). Furthermore, UVB-induced inhibition of the OEC, with an amplitude of up to ~24%, was significantly higher than that induced by UVA (~16%; *t*-test, *p*<0.05). In contrast, the PSII acceptor side activity ($\psi_{\mathrm{ET}2O}$; Figure 3A), quantum yield of electron transport (φ_Eo_; Figure 3B), PSI donor side activity ($\psi_{\mathrm{RE}1O}$; Figure 3C), and quantum yield for reduction of PSI acceptor side (φ_Ro_; Figure 3D) were significantly increased by UVR, as shown here by the negative inhibition values (*t*-test, *p*<0.05 for these four parameters). However, under the low-CO_2_ conditions, the effective quantum yield of PSI showed no significant change (*t*-test, *p*=0.487; Figure 4) between PAR and PAR+UVR treatments, indicating that PSI activity was less affected by UVR. However, an increase in the re-reduction rate of P700^+^ showed that UVR significantly stimulated the activity of CET around PSI, especially in the presence of UVB, with increasing amplitude by up to ~17% (~7% for UVA and~10% for UVB, respectively; *t*-test, *p*<0.05 for both UVA and UVB treatments; Figure 5). Due to the fact that CET relates to electron transport rate in both PSII and PSI [as it could be calcultated by the difference between ETRI and ETRII (Yamori et al., 2011; Gao and Wang, 2012)], the asynchronous variation between PSI activity and CET was mainly attributed to the decrease of PSII photochemical efficiency. The active CET thus compensates for the loss of linear electron transport rate, maintaining a high efficiency of generating ATP. Analyses of the specific energy fluxes of PSII showed that UVR significantly inhibited the density of PSII reaction centers (RC/CSo), the absorbed photon flux (ABS), the trapping photon flux (TRo) and the electron transport flux (ETo; *t*-test, *p*<0.05 for these four parameters), while there was an up-regulation of the dissipated energy flux (DIo; *t*-test, *p*<0.05; Figure 6).

In the future-simulated high-CO_2_ conditions, leading also to ocean acidification (OA), PAR alone did not induce any significant changes in *W*_k_ and *F*_v_/*F*_m_ (*t*-test, *p*=0.378 and 0.523, respectively; Figures 1, 2), indicating that both OEC and PSII were unaffected. The enhancements of $\psi_{\mathrm{ET}2O}$ (Figure 3A), φ_Eo_ (Figure 3B), $\psi_{\mathrm{RE}1O}$ (Figure 3C), and φ_Ro_ (Figure 3D) suggested that more electrons were transferred through the intersystem electron carriers under OA (*t*-test, *p*<0.05 for these four parameters). Regarding the downstream electron transport chain, YI (Figure 4) and re-reduction rate of P700^+^ (Figure 5) increased by up to ~11% and~23%, respectively, implying an up-regulation in PSI and CET (*t*-test, *p*<0.05 for these two parameters). Changes of the specific energy fluxes of PSII indicated the efficiency of active PSII reaction centers were enhanced by OA (*t*-test, *p*<0.05; Figure 6).

A two-way ANOVA analysis showed that both CO_2_ concentration, UVR, and their interaction, significantly affected OEC, PSII, the intersystem electron transport and CET activities, but not always PSI (Table 2). Under the high CO_2_ condition, UVR-induced inhibition of both OEC and PSII photochemical efficiency significantly decreased, with UVA- and UVB-induced inhibition of OEC decreased from ~15% to ~9%, and from ~24% to ~11%, respectively (*t*-test, *p*<0.05 for both UVA and UVB treatment); that of PSII by UVA and UVB ranged from ~16% to ~5%, and from ~15% to ~10%, respectively (*t*-test, *p*<0.05 for both UVA and UVB treatment; Figures 1, 2). Although the extent of UVR-induced inhibition on $\psi_{\mathrm{ET}2O}$, φ_Eo_, $\psi_{\mathrm{RE}1O}$, and φ_Ro_ exhibited significant differences between low- and high-CO_2_ conditions (Figure 3), the absolute values of these parameters were less affected (*t*-test, *p*=0.647, 0.548, 0.398 and 0.712 respectively). The significant difference in P700^+^ re-reduction between low- and high-CO_2_ indicated that there was a synergistic effect between increased CO_2_/OA and UVR, the high-CO_2_ further enhanced CET activity by up to ~4% and~5% under the influences of UVA and UVB, respectively (*t*-test, *p*<0.05 for both UVA and UVB treatment; Table 2, Figure 5). In PSII, UVR-induced inhibition on the density of PSII reaction centers (RC/CSo), the absorbed photon flux (ABS), the trapping photon flux (TRo), and the electron transport flux (ETo) was alleviated by the high-CO_2_ treatment (*t*-test, *p*<0.05 for both UVA and UVB treatment; Figure 6). Meanwhile, UVR-induced up-regulation of dissipated energy flux (DIo) was further enhanced under the high-CO_2_/OA condition (*t*-test, *p*<0.05 for both UVA and UVB treatment; Figure 6).

**Table 2:** Two-way ANOVA for the effects of CO_2_ (~400 and~1,000 μatm) and irradiance quality photosynthetically active radiation (PAR, PAR+UVA and PAR+UVA+UVB) on the OEC activity (W_k_), photosystem II (PSII) photochemical efficiency (*F*_v_/*F*_m_), intersystem electron transport efficiencies ($\psi_{\mathrm{ET}2O}$, φ_Eo,_ $\psi_{\mathrm{RE}1O}$, φ_Ro_), photosystem I (PSI) activity (YI) and CET activity (P700^+^ re-reduction).

	Irradiance quality		CO_2_		Irradiance quality × CO_2_	
	F	*p*	F	*p*	F	*p*
OEC activity (W_k_)	45.06	<0.001	12.89	0.004	5.72	0.018
PSII photochemical efficiency (*F*_v_/*F*_m_)	354.51	<0.001	21.52	0.001	5.38	0.021
PSII acceptor side activity ($\psi_{\mathrm{ET}2O}$)	32.17	<0.001	34.66	<0.001	8.73	0.005
Quantum yield of electron transport (φ_Eo_)	7.33	0.008	49.49	<0.001	4.90	0.028
PSI donor side activity ($\psi_{\mathrm{RE}1O}$)	79.26	<0.001	13.24	0.003	5.28	0.023
Quantum yield for reduction of PSI acceptor side (φ_Ro_)	5.58	0.019	26.87	<0.001	4.39	0.037
Effective quantum yield of PSI (YI)	1.94	0.186	22.12	0.001	0.70	0.515
CET activity (P700^+^ re-reduction)	23.46	<0.001	146.14	<0.001	4.63	0.032

## Discussion

**Figure 7:**
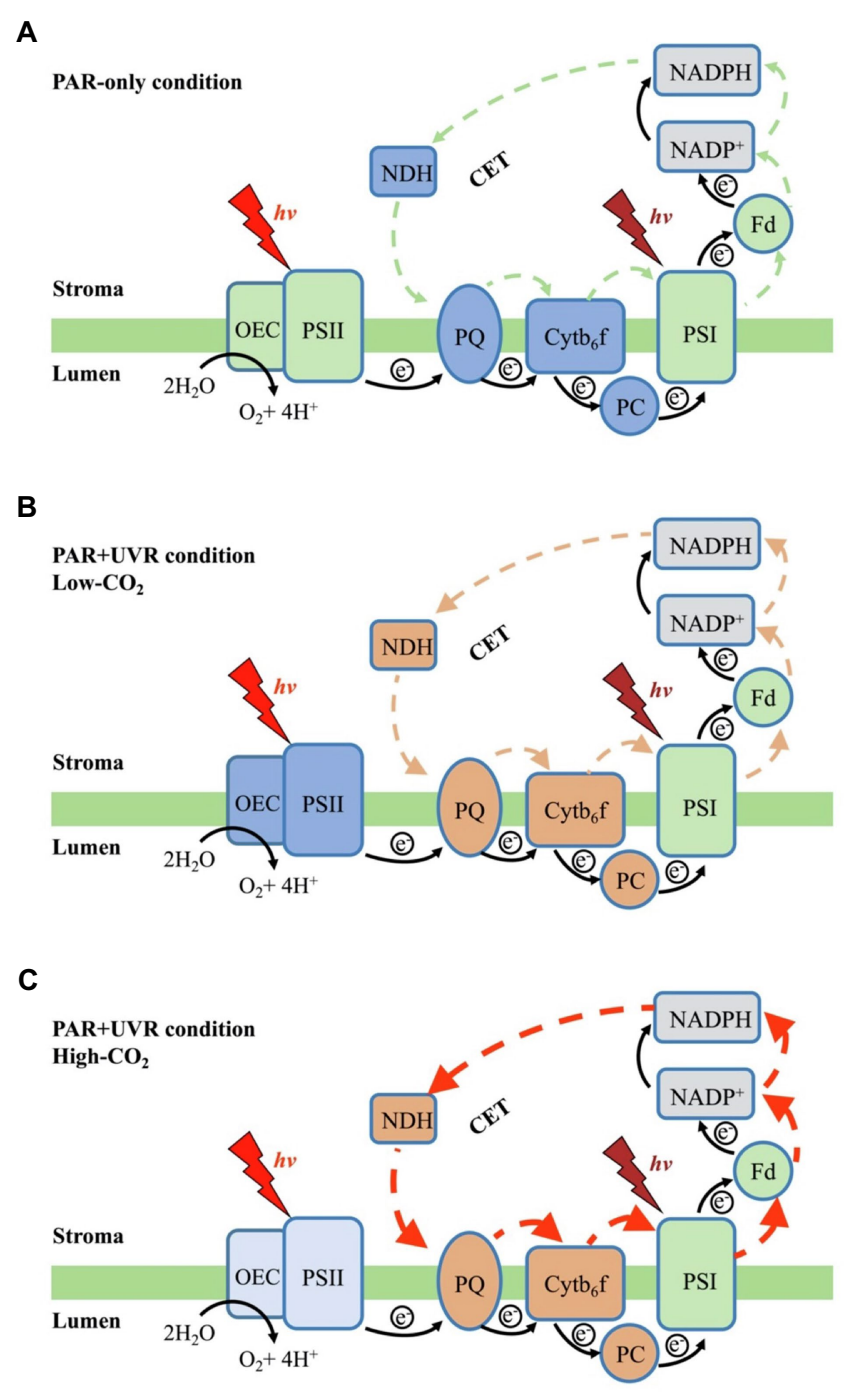
Schematic representation of the coordination in *P. yezoensis* photosynthetic electron transport when exposed to PAR-only **(A)**, or to PAR+UVR under low-CO_2_ **(B)** or high-CO_2_ **(C)** concentrations. The green area indicates a normal state, the dark blue areas indicated a more severe inhibition, the light blue indicates a moderate inhibition, the orange area indicates an up-regulation, and the gray areas represent the unmeasured sites. Black arrows indicate linear electron transport flow, the colored arrows CET around PSI and the orange/red arrows indicate the up-regulation of CET.

Our results suggest that in the red algae *P. yezoensis* (Ueda) M. S. Hwang and H. G. Choi, future elevated CO_2_ and ocean acidification (OA) can alleviate both UVB- and UVA-induced inhibition on PSII by modulating the synergy between PSII and PSI. Such synergy was found to relate mainly to the up-regulation of the intersystem electron transport efficiencies and CET around PSI (see Figure 7). In contrast with high-light-induced over-reduction of inter-photosystem electron transfer carriers (Figure 7A), UVR (especially UVB)-induced photoinhibition, characterized by the inhibition of OEC and PSII (Figure 7B), significantly decreased its quantum yield (Figure 2), which should be responsible for the reduced rates of carbon assimilation and growth (Figure 7B; Zhang et al., 2020). When grown and acclimated in the high-CO_2_ condition (Figure 7C), the well- established coordination between PSII and PSI, as well as the enhanced CET around PSI sustain the efficient electron transport, consequently increasing the resilience of *P. yezoensis* to PAR and/or UVR.

Previous studies have shown that the presence of UVR would reduce primary productivity in cyanobacteria and of phytoplankton assemblages by about 20% due to the concomitant photoinhibition (Helbling et al., 2003; Neale and Thomas, 2017; Williamson et al., 2019). In our previous study (Zhang et al., 2020), UVR-induced growth inhibition of *P. yezoensis* was ~31%, with only about 5% being attributable to UVB, implying that UVR-induced loss of carbon fixation was mainly driven by the negative effects of UVA. However, here, we show that both UVA and UVB significantly inhibited the OEC and PSII, and the presence of UVB markedly exacerbated photoinhibition by 24% for OEC and 15% for PSII (Figures 1, 2). Macroalgae have evolved several adaptive mechanisms to cope with photoinhibition, by increasing NPQ and UVACs(Gao and Xu, 2008; Zheng and Gao, 2009; Zhang et al., 2020), enhancing the xanthophyll cycle (Häder et al., 2002; Aigner et al., 2017; Xie et al., 2020) as well as antioxidant systems (Sureda et al., 2008; Li et al., 2010). In the present work, the responses of photosynthetic electron transport to UVR (UVA and UVB) and the related modulations between the photosystems are speculated to be responsible for the observed asymmetric responses between photoinhibition and growth.

Under the influence of UVR, the deactivation of OEC would lower the efficiency of water splitting, and thus the excess excitation energy would also result in an accumulation of ROS, as well as P680^+^ (Turcsányi and Vass, 2000; Tyystjärvi, 2008). These oxidized components can damage the D1 protein and lead to PSII photoinhibition (Zsiros et al., 2006). Our present study suggested that UVR inhibited the catalytic manganese cluster of the water-oxidizing complex, which has also been shown in other photosynthetic organisms (Vass et al., 1996; Turcsányi and Vass, 2000). Such damages are correlated with decreased O_2_ evolution in the tested species of *Pyropia* (Supplementary Table S1, Figueroa et al., 1997; Aguilera et al., 1999, 2008). Nevertheless, such photoinhibition could lower electron transport from PSII to PSI and thus protect the intersystem electron carriers and PSI from over-reduction and alleviating PSI from photoinhibition (Figures 4, 5, 7C; as suggested also by Larosa et al., 2018).

In view of the impacts of increased CO_2_ and OA, a number of previous studies have shown that high CO_2_/OA treatments did benefit O_2_ evolution and carbon assimilation in *Pyropia* spp. (Supplementary Table S2, Gao et al., 1991; Mercado et al., 1999; Chen et al., 2016, 2017; Zhang et al., 2020). Our results showed here that both OEC and PSII of *P. yezoensis* were less affected under PAR-only conditions, with PSI and CET being significantly up-regulated (Figures 4, 5). In contrast to the donor side photoinhibition induced by UVR, high-light induced photoinhibition is usually related to the over-reduction of intersystem electron carriers (Vass et al., 2005; Tyystjärvi, 2008). In the present work, the enhancement of CET would work as an alternative electron flow sink, together with the oxidized PSI, promoting the intersystem electron carriers to become oxidized, as reflected in negative inhibition (i.e., enhancement; Figure 3). Moreover, the up-regulated CET could also regulate the energy balance by consuming NADPH and generating ATP; NADP^+^ can also accept more electrons transferred from PSII and then oxidize the intersystem electron carriers (as suggested by Bukhov and Carpentier, 2004; Rumeau et al., 2007; Gao and Wang, 2012; Yu et al., 2018), thus contributing to the supply of energy for carboxylation.

The interactive effects of UVR and CO_2_ enrichment have been previously reported to be species-specific and UV-intensity-dependent (Gordillo et al., 2015; Ji and Gao, 2020 and references therein). A moderate UVR exposure amplified the positive effects of CO_2_ and OA on the red coralline algae *Corallina officinalis* under low PAR (Yildiz et al., 2013), while the synergistic effect of incident solar UVR and OA resulted in a decrease in both photosynthesis and calcification of the coralline algae *Corallina sessilis* (Gao and Zheng, 2010). In *P. yezoensis*, our results suggested that the increased CO_2_ and associated OA alleviated UVR-induced inhibition of the photosynthetic processes. Under the high-CO_2_ conditions, the up-regulation of CET would generate a higher trans-thylakoid proton gradient (ΔpH), which increase NPQ and could produce ATP for carbon assimilation. Moreover, the higher ΔpH-induced acidification of the lumen could also drive a Ca^2+^/H^+^ antiport to sequester Ca^2+^ into the lumen (Krieger and Weis, 1993; Ettinger et al., 1999), and thus aid in maintaining OEC stability, as reflected by our data showing alleviation of UVR-induced OEC inhibition by high CO_2_ treatments (Figure 1). Accordingly, OEC inhibition-induced photo-oxidative damage was significantly decreased, as evidenced by the increase of PSII photochemical efficiency (Figure 2), as well as the enhancement of the efficiency of the active PSII reaction center (Figure 6). Similar response was also observed in a tropical tree species (Huang et al., 2016) and marine angiosperm (Tan et al., 2020).

As mentioned above, the elevated DIC/CO_2_ in seawater can down-regulate the CCMs, which is also true for *P. yezoensis* (Li et al., 2016). However, little attention has been paid to the effects of high PAR/UVR as well as its combined effects with high CO_2_-induced OA. According to several published papers, high PAR/UVR affects the CCMs in different ways, and the effect is species-specific and light intensity dependent. For example, UVR enhanced the activity of extracellular carbonic anhydrase in *Skeletonema costatum* and thus enhanced its CCM (Wu and Gao, 2009), while a short-term exposure to UVR did not affect the inorganic carbon acquisition in *Dunaliella tertiolecta* (Beardall et al., 2002). Considering the fact that inorganic carbon acquisition is the prerequisite for carbon assimilation, which is the main photosynthetic electron sink, investigations of CCMs under high PAR/UVR and high CO_2_ are expected in future studies.

Under natural conditions in sea-farming areas, macroalgae experience low pH and high CO_2_ during the early morning period due to respiratory CO_2_ release at night. Our results imply that the red algae *P. yezoensis* can take advantage of the concomitant changes in the pCO_2_ and pH to cope with increasing UV exposure following sunrise. In addition, progressive OA associated with CO_2_ rise could positively enhance the alga’s photosynthesis and growth even under the influences of UVR, owing to the modulated synergy between PSII and PSI.

## Data Availability Statement

The raw data supporting the conclusions of this article will be made available by the authors, without undue reservation.

## Author Contributions

DZ: conceptualization, data collection and curation, data analysis, visualization, writing – original draft, and review and editing. JX: data collection and curation and review and editing. SB and JB: data analysis, formal analysis, and writing – review and editing. CZ: formal analysis and writing – review and editing. KG: conceptualization, funding acquisition, project administration, writing – original draft, and review and editing. All authors contributed to the article and approved the submitted version.

## Funding

This study was supported by the National Natural Science Foundation (41720104005, 41721005, and 41890803).

## Conflict of Interest

The authors declare that the research was conducted in the absence of any commercial or financial relationships that could be construed as a potential conflict of interest.

## Publisher’s Note

All claims expressed in this article are solely those of the authors and do not necessarily represent those of their affiliated organizations, or those of the publisher, the editors and the reviewers. Any product that may be evaluated in this article, or claim that may be made by its manufacturer, is not guaranteed or endorsed by the publisher.

## References

[ref1] Aguilera J. Figueroa F. L. Häder D. P. Jiménez C. (2008). Photoinhibition and photosynthetic pigment reorganisation dynamics in light/darkness cycles as photoprotective mechanisms of *Porphyra umbilicalis* against damaging effects of UV radiation. Sci. Mar. 72, 87–97. doi: 10.3989/scimar.2008.72n187

[ref2] Aguilera J. Jiménez C. Figueroa F. L. Lebert M. Häder D. P. (1999). Effect of ultraviolet radiation on thallus absorption and photosynthetic pigments in the red alga *Porphyra umbilicalis*. J. Photochem. Photobiol. B 48, 75–82. doi: 10.1016/S1011-1344(99)00015-9

[ref3] Aigner S. Holzinger A. Karsten U. Kranner I. (2017). The freshwater red alga *Batrachospermum turfosum* (Florideophyceae) can acclimate to a wide range of light and temperature conditions. Eur. J. Phycol. 52, 238–249. doi: 10.1080/09670262.2016.1274430, PMID: 28413232 PMC5390863

[ref4] Aline T. Atkinson M. J. Christopher L. (2006). Effects of elevated pCO_2_ on epilithic and endolithic metabolism of reef carbonates. Glob. Chang. Biol. 12, 2200–2208. doi: 10.1111/j.1365-2486.2006.01249.x

[ref5] Andría J. R. Brun F. G. Pérez-Lloréns J. L. Vergara J. J. (2001). Acclimation responses of *Gracilaria* sp. (Rhodophyta) and *Enteromorpha intestinalis* (Chlorophyta) to changes in the external inorganic carbon concentration. Bot. Mar. 44, 361–370. doi: 10.1515/BOT.2001.046

[ref6] Andría J. Vergara J. Pérez-Lloréns J. L. (1999). Biochemical responses and photosynthetic performance of *Gracilaria* sp. (Rhodophyta) from Cádiz, Spain, cultured under different inorganic carbon and nitrogen levels. Eur. J. Phycol. 34, 497–504.

[ref7] Beardall J. Heraud P. Roberts S. Shelly K. Stojkovic S. (2002). Effects of UV-B radiation on inorganic carbon acquisition by the marine microalga *Dunaliella tertiolecta* (Chlorophyceae). Phycologia 41, 268–272. doi: 10.2216/i0031-8884-41-3-268.1

[ref8] Beer S. Koch E. (1996). Photosynthesis of marine macroalgae and seagrasses in globally changing CO_2_ environments. Mar. Ecol. Prog. Ser. 141, 199–204. doi: 10.3354/meps141199

[ref9] Blouin N. A. Brodie J. A. Grossman A. C. Xu P. Brawley S. H. (2011). *Porphyra*: a marine crop shaped by stress. Trends Plant Sci. 16, 29–37. doi: 10.1016/j.tplants.2010.10.004, PMID: 21067966

[ref10] Büdenbender J. Riebesell U. Form A. (2011). Calcification of the Arctic coralline red algae *Lithothamnion glaciale* in response to elevated CO_2_. Mar. Ecol. Prog. Ser. 441, 79–87. doi: 10.3354/meps09405

[ref11] Bukhov N. Carpentier R. (2004). Alternative photosystem I-driven electron transport routes: mechanisms and functions. Photosynth. Res. 82, 17–33. doi: 10.1023/B:PRES.0000040442.59311.72, PMID: 16228610

[ref12] Chen B. Zou D. Ma J. (2016). Interactive effects of elevated CO_2_ and nitrogen–phosphorus supply on the physiological properties of *Pyropia haitanensis* (Bangiales, Rhodophyta). J. Appl. Phycol. 28, 1235–1243. doi: 10.1007/s10811-015-0628-z

[ref13] Chen B. Zou D. Yang Y. (2017). Increased iron availability resulting from increased CO_2_ enhances carbon and nitrogen metabolism in the economical marine red macroalga *Pyropia haitanensis* (Rhodophyta). Chemosphere 173, 444–451. doi: 10.1016/j.chemosphere.2017.01.073, PMID: 28131089

[ref14] Eberhard S. Finazzi G. Wollman F. A. (2008). The dynamics of photosynthesis. Annu. Rev. Genet. 42, 463–515. doi: 10.1146/annurev.genet.42.110807.091452, PMID: 18983262

[ref15] Ettinger W. F. Clear A. M. Fanning K. J. Peck M. L. (1999). Identification of a Ca^2+^/H^+^ antiport in the plant chloroplast thylakoid membrane. Plant Physiol. 119, 1379–1386. doi: 10.1104/pp.119.4.1379, PMID: 10198097 PMC32023

[ref16] Figueroa F. L. Salles S. Aguilera J. Jiménez C. Mercado J. Viñegla B.. (1997). Effects of solar radiation on photoinhibition and pigmentation in the red alga *Porphyra leucosticta*. Mar. Ecol. Prog. Ser. 151, 81–90. doi: 10.3354/meps151081

[ref17] Gao K. Aruga Y. Asada K. Ishihara T. Akano T. Kiyohara M. (1991). Enhanced growth of the red alga *Porphyra yezoensis* Ueda in high CO_2_ concentrations. J. Appl. Phycol. 3, 355–362. doi: 10.1007/BF02392889

[ref18] Gao K. Aruga Y. Asada K. Ishihara T. Akano T. Kiyohara M. (1993). Calcification in the articulated coralline alga *Corallina pilulifera*, with special reference to the effect of elevated CO_2_ concentration. Mar. Biol. 117, 129–132. doi: 10.1007/BF00346434

[ref19] Gao K. Beardall J. Häder D. P. Hall-Spencer J. M. Gao G. Hutchins D. A. (2019). Effects of ocean acidification on marine photosynthetic organisms under the concurrent influences of warming, UV radiation, and deoxygenation. Front. Mar. Sci. 6:322. doi: 10.3389/fmars.2019.00322

[ref20] Gao K. Guan W. Helbling E. W. (2007). Effects of solar ultraviolet radiation on photosynthesis of the marine red tide alga *Heterosigma akashiwo* (Raphidophyceae). J. Photochem. Photobiol. B 86, 140–148. doi: 10.1016/j.jphotobiol.2006.05.007, PMID: 17045485

[ref21] Gao S. Niu J. Chen W. Wang G. Xie X. Pan G.. (2013). The physiological links of the increased photosystem II activity in moderately desiccated *Porphyra haitanensis* (Bangiales, Rhodophyta) to the cyclic electron flow during desiccation and re-hydration. Photosynth. Res. 116, 45–54. doi: 10.1007/s11120-013-9892-4, PMID: 23896795

[ref22] Gao S. Wang G. (2012). The enhancement of cyclic electron flow around photosystem I improves the recovery of severely desiccated *Porphyra yezoensis* (Bangiales, Rhodophyta). J. Exp. Bot. 63, 4349–4358. doi: 10.1093/jxb/ers082, PMID: 22438301

[ref23] Gao K. Xu J. (2008). Effects of solar UV radiation on diurnal photosynthetic performance and growth of *Gracilaria lemaneiformis* (Rhodophyta). Eur. J. Phycol. 43, 297–307. doi: 10.1080/09670260801986837

[ref24] Gao K. Zheng Y. (2010). Combined effects of ocean acidification and solar UV radiation on photosynthesis, growth, pigmentation and calcification of the coralline alga *Corallina sessilis* (Rhodophyta). Glob. Chang. Biol. 16, 2388–2398. doi: 10.1111/j.1365-2486.2009.02113.x

[ref25] Gordillo F. J. Aguilera J. Wiencke C. Jiménez C. (2015). Ocean acidification modulates the response of two Arctic kelps to ultraviolet radiation. J. Plant Physiol. 173, 41–50. doi: 10.1016/j.jplph.2014.09.008, PMID: 25462077

[ref26] Guisse B. Srivastava A. Strasser R. (1995). The polyphasic rise of the chlorophyll *a* fluorescence (OKJIP) in heat-stressed leaves. Arch. Sci. 48, 147–160. doi: 10.5169/SEALS-740252

[ref27] Häder D. P. Barnes P. W. (2019). Comparing the impacts of climate change on the responses and linkages between terrestrial and aquatic ecosystems. Sci. Total Environ. 682, 239–246. doi: 10.1016/j.scitotenv.2019.05.024, PMID: 31121350

[ref28] Häder D. P. Lebert M. Sinha R. P. Barbieri E. S. Helbling E. W. (2002). Role of protective and repair mechanisms in the inhibition of photosynthesis in marine macroalgae. Photochem. Photobiol. Sci. 1, 809–814. doi: 10.1039/B206152J, PMID: 12656483

[ref29] Helbling E. W. Gao K. Gonçalves R. J. Wu H. Villafañe V. E. (2003). Utilization of solar UV radiation by coastal phytoplankton assemblages off SE China when exposed to fast mixing. Mar. Ecol. Prog. Ser. 259, 59–66. doi: 10.3354/meps259059

[ref30] Huang W. Yang Y. J. Hu H. Zhang S. B. Cao K. F. (2016). Evidence for the role of cyclic electron flow in photoprotection for oxygen-evolving complex. J. Plant Physiol. 194, 54–60. doi: 10.1016/j.jplph.2016.02.016, PMID: 26968082

[ref31] Hurd C. L. Beardall J. Comeau S. Cornwall C. E. Havenhand J. N. Munday P. L.. (2020). Ocean acidification as a multiple driver: how interactions between changing seawater carbonate parameters affect marine life. Mar. Freshw. Res. 71, 263–274. doi: 10.1071/MF19267

[ref32] IPCC (2014) in Climate Change 2014: Synthesis Report. Contribution of Working Groups I, II and III to the Fifth Assessment Report of the Intergovernmental Panel on Climate Change. eds. Core Writing Team, Pachauri R. K. Meyer L. A. (Geneva, Switzerland: IPCC), 151.

[ref33] Ji Y. Gao K. (2020). Effects of climate change factors on marine macroalgae: a review. Adv. Mar. Biol. 88, 91–136. doi: 10.1016/bs.amb.2020.11.001, PMID: 34119047

[ref34] Jiang H. Gao K. Helbling E. W. (2007). Effects of solar UV radiation on germination of conchospores and morphogenesis of sporelings in *Porphyra haitanensis* (Rhodophyta). Mar. Biol. 151, 1751–1759. doi: 10.1007/s00227-007-0632-1

[ref35] Klughammer C. Schreiber U. (1994). An improved method, using saturating light pulses, for the determination of photosystem I quantum yield via P700^+^-absorbance changes at 830 nm. Planta 192, 261–268. doi: 10.1007/BF01089043

[ref36] Korbee N. Navarro N. P. García-Sánchez M. Celis-Plá P. S. M. Quintano E. Copertino M. D. S.. (2014). A novel in situ system to evaluate the effect of high CO_2_ on photosynthesis and biochemistry of seaweeds. Aquat. Biol. 22, 245–259. doi: 10.3354/ab00594

[ref37] Krieger A. Weis E. (1993). The role of calcium in the pH-dependent control of photosystem II. Photosynth. Res. 37, 117–130. doi: 10.1007/BF02187470, PMID: 24317708

[ref38] Larosa V. Meneghesso A. La Rocca N. Steinbeck J. Hippler M. Szabò I.. (2018). Mitochondria affect photosynthetic electron transport and photosensitivity in a green alga. Plant Physiol. 176, 2305–2314. doi: 10.1104/pp.17.01249, PMID: 29284743 PMC5841685

[ref39] Li X. Xu J. He P. (2016). Comparative research on inorganic carbon acquisition by the macroalgae *Ulva prolifera* (Chlorophyta) and *Pyropia yezoensis* (Rhodophyta). J. Appl. Phycol. 28, 491–497. doi: 10.1007/s10811-015-0603-8

[ref40] Li L. Zhao J. Tang X. (2010). Ultraviolet irradiation induced oxidative stress and response of antioxidant system in an intertidal macroalgae *Corallina officinalis* L. J. Environ. Sci. 22, 716–722. doi: 10.1016/S1001-0742(09)60168-6, PMID: 20608508

[ref100] Lu X. Huan L. Gao S. Gao S. (2016). NADPH from the oxidative pentose phosphate pathway drives the operation of cyclic electron flow around photosystem I in high-intertidal macroalgae under severe salt stress *Physiol*. Plantarum. 156, 397–406. doi: 10.1111/ppl.12383, PMID: 26337725

[ref41] Martin S. Gattuso J. P. (2009). Response of Mediterranean coralline algae to ocean acidification and elevated temperature. Glob. Chang. Biol. 15, 2089–2100. doi: 10.1111/j.1365-2486.2009.01874.x

[ref42] Mercado J. M. Javier F. Gordillo L. Niell F. X. Figueroa F. L. (1999). Effects of different levels of CO_2_ on photosynthesis and cell components of the red alga *Porphyra leucosticta*. J. Appl. Phycol. 11, 455–461. doi: 10.1023/A:1008194223558

[ref43] Miyake C. (2010). Alternative electron flows (water–water cycle and cyclic electron flow around PSI) in photosynthesis: molecular mechanisms and physiological functions. Plant Cell Physiol. 51, 1951–1963. doi: 10.1093/pcp/pcq173, PMID: 21068108

[ref44] Neale R. E. Barnes P. W. Robson T. M. Neale P. J. Williamson C. E. Zepp R. G.. (2021). Environmental effects of stratospheric ozone depletion, UV radiation, and interactions with climate change: UNEP environmental effects assessment panel, update 2020. Photochem. Photobiol. Sci. 20, 1–67. doi: 10.1007/s43630-020-00001-x, PMID: 33721243 PMC7816068

[ref45] Neale P. J. Thomas B. C. (2017). Inhibition by ultraviolet and photosynthetically available radiation lowers model estimates of depth-integrated picophytoplankton photosynthesis: global predictions for *Prochlorococcus* and *Synechococcus*. Glob. Chang. Biol. 23, 293–306. doi: 10.1111/gcb.13356, PMID: 27178715

[ref46] Neubauer C. Schreiber U. (1987). The polyphasic rise of chlorophyll fluorescence upon onset of strong continuous illumination: I. saturation characteristics and partial control by the photosystem II acceptor side. Zeitschrift für Naturforschung C 42, 1246–1254. doi: 10.1515/znc-1987-11-1217

[ref47] Niu J. Feng J. Xie X. Gao S. Wang G. (2016). Involvement of cyclic electron flow in irradiance stress responding and its potential regulation of the mechanisms in *Pyropia yezoensis*. Chin. J. Oceanol. Limnol. 34, 730–739. doi: 10.1007/s00343-016-4236-9

[ref48] Pierrot D. Lewis E. Wallace D. W. R. (2006). MS Excel program developed for CO_2_ system calculations. ORNL/CDIAC-105a. Environ. Sci. doi: 10.3334/CDIAC/otg.CO2SYS_XLS_CDIAC105a

[ref49] Raven J. A. Beardall J. Giordano M. (2014). Energy costs of carbon dioxide concentrating mechanisms in aquatic organisms. Photosynth. Res. 121, 111–124. doi: 10.1007/s11120-013-9962-7, PMID: 24390639

[ref50] Rumeau D. Peltier G. Cournac L. (2007). Chlororespiration and cyclic electron flow around PSI during photosynthesis and plant stress response. Plant Cell Environ. 30, 1041–1051. doi: 10.1111/j.1365-3040.2007.01675.x, PMID: 17661746

[ref51] Semesi I. S. Kangwe J. Björk M. (2009). Alterations in seawater pH and CO_2_ affect calcification and photosynthesis in the tropical coralline alga, *Hydrolithon* sp. (Rhodophyta). Estuar. Coast. Shelf Sci. 84, 337–341. doi: 10.1016/j.ecss.2009.03.038

[ref52] Strasser R. J. (1981). “The grouping model of plant photosynthesis: heterogeneity of photosynthetic units in thylakoids,” in Photosynthesis III. Structure and Molecular Organisation of the Photosynthetic Apparatus. ed. Akoyonoglou G. (Philadelphia: Balaban International Science Services), 727–737.

[ref53] Strasser B. J. Strasser R. J. (1995). “Measuring fast fluorescence transients to address environmental questions: The JIP-test,” in Photosynthesis: From Light to Biosphere. ed. Mathis. Vol. 5 ed (Netherlands: Kluwer Academic Publishers), 977–980.

[ref54] Strasser R. J. Tsimilli-Michael M. Srivastava A. (2004). “Analysis of the chlorophyll *a* fluorescence transient,” in Chlorophyll a Fluorescence. eds. Papageorgiou G. C. Govindjee (Dordrecht: Springer), 321–362.

[ref55] Su H. N. Xie B. B. Zhang X. Y. Zhou B. C. Zhang Y. Z. (2010). The supramolecular architecture, function, and regulation of thylakoid membranes in red algae: an overview. Photosynth. Res. 106, 73–87. doi: 10.1007/s11120-010-9560-x, PMID: 20521115

[ref56] Suárez-Álvarez S. Gómez-Pinchetti J. L. García-Reina G. (2012). Effects of increased CO_2_ levels on growth, photosynthesis, ammonium uptake and cell composition in the macroalga *Hypnea spinella* (Gigartinales, Rhodophyta). J. Appl. Phycol. 24, 815–823. doi: 10.1007/s10811-011-9700-5

[ref57] Sureda A. Box A. Terrados J. Deudero S. Pons A. (2008). Antioxidant response of the seagrass Posidonia oceanica when epiphytized by the invasive macroalgae *Lophocladia lallemandii*. Mar. Environ. Res. 66, 359–363. doi: 10.1016/j.marenvres.2008.05.009, PMID: 18639925

[ref58] Tan Y. Zhang Q. S. Zhao W. Liu Z. Ma M. Y. Zhong M. Y.. (2020). The highly efficient NDH-dependent photosystem I cyclic electron flow pathway in the marine angiosperm *Zostera marina*. Photosynth. Res. 144, 49–62. doi: 10.1007/s11120-020-00732-z, PMID: 32152819

[ref59] Turcsányi E. Vass I. (2000). Inhibition of photosynthetic electron transport by UV-A radiation targets the photosystem II complex. Photochem. Photobiol. 72, 513–520. doi: 10.1562/0031-8655(2000)072<0513:IOPETB>2.0.CO;2, PMID: 11045723

[ref60] Tyystjärvi E. (2008). Photoinhibition of photosystem II and photodamage of the oxygen evolving manganese cluster. Coord. Chem. Rev. 252, 361–376. doi: 10.1016/j.ccr.2007.08.021

[ref61] Vass I. Sass L. Spetea C. Bakou A. Ghanotakis D. F. Petrouleas V. (1996). UV-B-induced inhibition of photosystem II electron transport studied by EPR and chlorophyll fluorescence. Impairment of donor and acceptor side components. Biochemistry 35, 8964–8973. doi: 10.1021/bi9530595, PMID: 8688433

[ref62] Vass I. Szilárd A. Sicora C. (2005). “Adverse effects of UV-B light on the structure and function of the photosynthetic apparatus,” in Handbook of Photosynthesis. ed. Mohammad Pessarakli (Boca Raton, FL, USA: Francis and Taylor publisher), 43–63.

[ref63] Viñegla B. Segovia M. Figueroa F. L. (2006). Effect of artificial UV radiation on carbon and nitrogen metabolism in the macroalgae *Fucus spiralis* L. and *Ulva olivascens* Dangeard. Hydrobiologia 560, 31–42. doi: 10.1007/s10750-005-1097-1

[ref64] Williamson C. E. Neale P. J. Hylander S. Rose K. C. Figueroa F. L. Robinson S. A.. (2019). The interactive effects of stratospheric ozone depletion, UV radiation, and climate change on aquatic ecosystems. Photochem. Photobiol. Sci. 18, 717–746. doi: 10.1039/c8pp90062k, PMID: 30810561

[ref65] Wu H. Gao K. (2009). Ultraviolet radiation stimulated activity of extracellular carbonic anhydrase in the marine diatom *Skeletonema costatum*. Funct. Plant Biol. 36, 137–143. doi: 10.1071/FP08172, PMID: 32688633

[ref66] Xie X. Lu X. Wang L. He L. Wang G. (2020). High light intensity increases the concentrations of β-carotene and zeaxanthin in marine red macroalgae. Algal Res. 47:101852. doi: 10.1016/j.algal.2020.101852

[ref67] Xu J. Gao K. (2008). Growth, pigments, UV-absorbing compounds and agar yield of the economic red seaweed *Gracilaria lemaneiformis* (Rhodophyta) grown at different depths in the coastal waters of the South China Sea. J. Appl. Phycol. 20, 681–686. doi: 10.1007/s10811-007-9247-7

[ref68] Xu J. Gao K. (2010). UV-A enhanced growth and UV-B induced positive effects in the recovery of photochemical yield in *Gracilaria lemaneiformis* (Rhodophyta). J. Photochem. Photobiol. B 100, 117–122. doi: 10.1016/j.jphotobiol.2010.05.010, PMID: 20573516

[ref69] Xu J. Gao K. (2012). Future CO_2_-induced ocean acidification mediates the physiological performance of a green tide alga. Plant Physiol. 160, 1762–1769. doi: 10.1104/pp.112.206961, PMID: 23129205 PMC3510108

[ref70] Yamori W. Sakata N. Suzuki Y. Shikanai T. Makino A. (2011). Cyclic electron flow around photosystem I via chloroplast NAD (P) H dehydrogenase (NDH) complex performs a significant physiological role during photosynthesis and plant growth at low temperature in rice. Plant J. 68, 966–976. doi: 10.1111/j.1365-313X.2011.04747.x, PMID: 21848656

[ref71] Yildiz G. Hofmann L. C. Bischof K. Dere Ş. (2013). Ultraviolet radiation modulates the physiological responses of the calcified rhodophyte *Corallina officinalis* to elevated CO_2_. Bot. Mar. 56, 161–168. doi: 10.1515/bot-2012-0216

[ref72] Yu B. Niu J. Feng J. Xu M. Xie X. Gu W.. (2018). Regulation of ferredoxin-NADP^+^ oxidoreductase to cyclic electron transport in high salinity stressed *Pyropia yezoensis*. Front. Plant Sci. 9:1092. doi: 10.3389/fpls.2018.01092, PMID: 30090109 PMC6068275

[ref73] Zhang D. Xu J. Bao M. Yan D. Beer S. Beardall J.. (2020). Elevated CO_2_ concentration alleviates UVR-induced inhibition of photosynthetic light reactions and growth in an intertidal red macroalga. J. Photochem. Photobiol. B 213:112074. doi: 10.1016/j.jphotobiol.2020.112074, PMID: 33152637

[ref74] Zheng Y. Gao K. (2009). Impacts of solar UV radiation on the photosynthesis, growth, and UV-absorbing compounds in *Gracilaria lemaneiformis* (Rhodophyta) grown at different nitrate concentrations. J. Phycol. 45, 314–323. doi: 10.1111/j.1529-8817.2009.00654.x, PMID: 27033810

[ref75] Zou D. (2005). Effects of elevated atmospheric CO_2_ on growth, photosynthesis and nitrogen metabolism in the economic brown seaweed, *Hizikia fusiforme* (Sargassaceae, Phaeophyta). Aquaculture 250, 726–735. doi: 10.1016/j.aquaculture.2005.05.014

[ref76] Zou D. Gao K. (2005). Ecophysiological characteristics of four intertidal marine macroalgae during emersion along Shantou coast of China, with a special reference to the relationship of photosynthesis and CO_2_. Acta Oceanol. Sin. 24, 105–113.

[ref77] Zou D. Gao K. Xia J. (2003). Photosynthetic utilization of inorganic carbon in the economic brown alga, *Hizikia Fusiforme* (Sargassaceae) from the South China Sea. J. Phycol. 39, 1095–1100. doi: 10.1111/j.0022-3646.2003.03-038.x

[ref78] Zsiros O. Allakhverdiev S. I. Higashi S. Watanabe M. Nishiyama Y. Murata N. (2006). Very strong UV-A light temporally separates the photoinhibition of photosystem II into light-induced inactivation and repair. Biochim. Biophys. Acta 1757, 123–129. doi: 10.1016/j.bbabio.2006.01.004, PMID: 16500615

