# Supplementary material for: Increased CO2 Relevant to Future Ocean Acidification Alleviates the Sensitivity of a Red Macroalgae to Solar Ultraviolet Irradiance by Modulating the Synergy Between Photosystems II and I
Source: Front Plant Sci. 2021 Sep 16;12:726538. doi: 10.3389/fpls.2021.726538 (PMC8481898; doi:10.3389/fpls.2021.726538)
Supplement: Supplementary file 1 [file Data_Sheet_1.docx]

Fig. S1 Diagram for linear electron transfer from water to NADP^+^ plotted on a typically Kausky curve. The letters O, K, J, I and P refer to the selected time points used by the JIP-test for the calculation of structural and functional parameters. Modified from Govindjee (The 1^st^ Asia-Oceania International Congress on Photosynthesis, Beijing, 2018) and Kai Wang (Hansha Scientific Instruments).


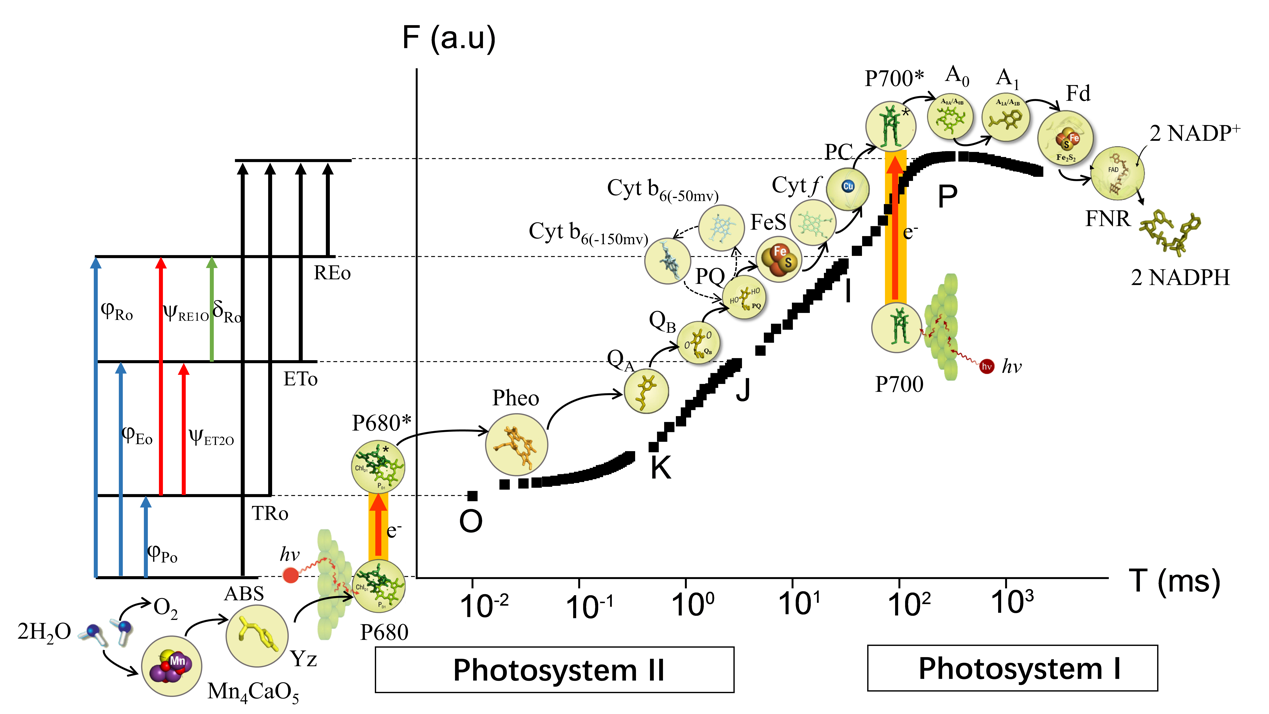


Table S1 Effects of PAR and PAR+UVR on O_2_ evolution rate of *Porphyra* spp.

| Species | Light treatments | CO_2_ treatments | Other conditions | O_2_ evolution rate | References |
| --- | --- | --- | --- | --- | --- |
| *Porphyra leucosticta* | PAR | Natural seawater | - | ~0.75 μmol O_2_ g FW^-1^ h^-1^  (Daily average) | Figueroa et al., 1997 |
|  | PAR+UVR |  |  | ~0.55μmol O_2_ g FW^-1^ h^-1^  (Daily average) |  |
| *Porphyra umbilicalis* | PAR | Natural seawater | High-phycobiliprotein-content thalli | ~0.9 mg O_2_ g FW^-1^ min^-1^ | Aguilera et al., 1999 |
|  | PAR+UVR |  |  | ~0.42 mg O_2_ g FW^-1^ min^-1^ |  |
|  | PAR |  | Low-phycobiliprotein-content thalli | ~0.65 mg O_2_ g FW^-1^ min^-1^ |  |
|  | PAR+UVR |  |  | ~0.45 mg O_2_ g FW^-1^ min^-1^ |  |
| *Porphyra umbilicalis* | PAR | Natural seawater | Continuous illumination | ~0.6 mg O_2_ g FW^-1^ min^-1^ | Aguilera et al., 2008 |
|  | PAR+UVR |  |  | ~0.3 mg O_2_ g FW^-1^ min^-1^ |  |
|  | PAR |  | 12:12 light: dark cycle | ~0.6 mg O_2_ g FW^-1^ min^-1^ |  |
|  | PAR+UVR |  |  | ~0.45 mg O_2_ g FW^-1^ min^-1^ |  |

Table S2 Effects of CO_2_ concentration variations on O_2_ evolution rate of *Porphyra* spp.

| Species | Light treatments | CO_2_ treatments | Other conditions | O_2_ evolution rate | References |
| --- | --- | --- | --- | --- | --- |
| *Porphyra yezoensis* | PAR | Natural seawater aerated with ~350 ppmv CO_2_ | 2 mM DIC | ~1.8μmol O_2_ cm^-2^ h^-1^ | Gao et al., 1991 |
|  |  | Natural seawater aerated with ~1000 ppmv CO_2_ |  | ~2.1μmol O_2_ cm^-2^ h^-1^ |  |
|  |  | Natural seawater aerated with ~1600 ppmv CO_2_ |  | ~1.8 μmol O_2_ cm^-2^ h^-1^ |  |
|  |  | Natural seawater aerated with ~350 ppmv CO_2_ | 10 mM DIC | ~2.35μmol O_2_ cm^-2^ h^-1^ |  |
|  |  | Natural seawater aerated with ~1000 ppmv CO_2_ |  | ~2.4μmol O_2_ cm^-2^ h^-1^ |  |
|  |  | Natural seawater aerated with ~1600 ppmv CO_2_ |  | ~2.5μmol O_2_ cm^-2^ h^-1^ |  |
| *Porphyra leucosticta* | PAR | Natural seawater aerated with ~<0.0001% CO_2_ (~6μmol DIC) | - | 227±23 μmol O_2_ g FW^-1^ h^-1^ (P_max_) | Mercado et al., 1999 |
|  |  | Natural seawater aerated with 0.035% CO_2_  (~2.5 mM DIC) |  | 290±30 μmol O_2_ g FW^-1^ h^-1^ (P_max_) |  |
|  |  | Natural seawater aerated with 0.035% CO_2_  (~0.2 M DIC) |  | 335±17 μmol O_2_ g FW^-1^ h^-1^ (P_max_) |  |

Table S2 continued

| *Porphyra haitanensis* | PAR | Natural seawater aerated with ~390 ppmv CO_2_ | ≤ 40 μmol N  < 1 μmol P | 50.71±4.14 μmol O_2_ g FW^-1^ h^-1^ (P_max_) | Chen et al., 2016 |
| --- | --- | --- | --- | --- | --- |
|  |  | Natural seawater aerated with ~1000 ppmv CO_2_ |  | 55.82±1.68 μmol O_2_ g FW^-1^ h^-1^ (P_max_) |  |
|  |  | Natural seawater aerated with ~390 ppmv CO_2_ | 300 μmol N  15 μmol P | 67.33±1.63 μmol O_2_ g FW^-1^ h^-1^ (P_max_) |  |
|  |  | Natural seawater aerated with ~1000 ppmv CO_2_ |  | 82.66±4.32 μmol O_2_ g FW^-1^ h^-1^ (P_max_) |  |
|  |  | Natural seawater aerated with ~390 ppmv CO_2_ | 600 μmol N  30 μmol P | 68.8±2.73 μmol O_2_ g FW^-1^ h^-1^ (P_max_) |  |
|  |  | Natural seawater aerated with ~1000 ppmv CO_2_ |  | 78.36±4.14 μmol O_2_ g FW^-1^ h^-1^ (P_max_) |  |
| *Porphyra haitanensis* | PAR | Natural seawater aerated with ~390 ppmv CO_2_ | Non-enriched iron | ~86 μmol O_2_ g FW^-1^ h^-1^ (P_max_) | Chen et al., 2017 |
|  |  | Natural seawater aerated with ~1000 ppmv CO_2_ |  | ~93 μmol O_2_ g FW^-1^ h^-1^ (P_max_) |  |
|  |  | Natural seawater aerated with ~390 ppmv CO_2_ | Enriched iron | ~92 μmol O_2_ g FW^-1^ h^-1^ (P_max_) |  |
|  |  | Natural seawater aerated with ~1000 ppmv CO_2_ |  | ~107 μmol O_2_ g FW^-1^ h^-1^ (P_max_) |  |
